# Cysteine catabolism and the serine biosynthesis pathway support pyruvate production during pyruvate kinase knockdown in pancreatic cancer cells

**DOI:** 10.1186/s40170-019-0205-z

**Published:** 2019-12-30

**Authors:** Lei Yu, Shao Thing Teoh, Elliot Ensink, Martin P. Ogrodzinski, Che Yang, Ana I. Vazquez, Sophia Y. Lunt

**Affiliations:** 1 Department of Biochemistry and Molecular Biology, Michigan State University, East Lansing, MI USA; 2 Department of Physiology, Michigan State University, East Lansing, MI USA; 3 Department of Epidemiology and Biostatistics, Michigan State University, East Lansing, MI USA; 4 The Institute for Quantitative Health Science and Engineering, Michigan State University, East Lansing, MI USA; 5 Department of Chemical Engineering and Materials Science, Michigan State University, East Lansing, MI USA

**Keywords:** Pyruvate kinase, PKM, Pancreatic cancer, Liquid chromatography mass spectrometry, Metabolism

## Abstract

**Background:**

Pancreatic ductal adenocarcinoma (PDAC) is an aggressive cancer with limited treatment options. Pyruvate kinase, especially the M2 isoform (PKM2), is highly expressed in PDAC cells, but its role in pancreatic cancer remains controversial. To investigate the role of pyruvate kinase in pancreatic cancer, we knocked down PKM2 individually as well as both PKM1 and PKM2 concurrently (PKM1/2) in cell lines derived from a *Kras*^*G12D/-*^*; p53*^*-/-*^ pancreatic mouse model.

**Methods:**

We used liquid chromatography tandem mass spectrometry (LC-MS/MS) to determine metabolic profiles of wildtype and PKM1/2 knockdown PDAC cells. We further used stable isotope-labeled metabolic precursors and LC-MS/MS to determine metabolic pathways upregulated in PKM1/2 knockdown cells. We then targeted metabolic pathways upregulated in PKM1/2 knockdown cells using CRISPR/Cas9 gene editing technology.

**Results:**

PDAC cells are able to proliferate and continue to produce pyruvate despite PKM1/2 knockdown. The serine biosynthesis pathway partially contributed to pyruvate production during PKM1/2 knockdown: knockout of phosphoglycerate dehydrogenase in this pathway decreased pyruvate production from glucose. In addition, cysteine catabolism generated ~ 20% of intracellular pyruvate in PDAC cells. Other potential sources of pyruvate include the sialic acid pathway and catabolism of glutamine, serine, tryptophan, and threonine. However, these sources did not provide significant levels of pyruvate in PKM1/2 knockdown cells.

**Conclusion:**

PKM1/2 knockdown does not impact the proliferation of pancreatic cancer cells. The serine biosynthesis pathway supports conversion of glucose to pyruvate during pyruvate kinase knockdown. However, direct conversion of serine to pyruvate was not observed during PKM1/2 knockdown. Investigating several alternative sources of pyruvate identified cysteine catabolism for pyruvate production during PKM1/2 knockdown. Surprisingly, we find that a large percentage of intracellular pyruvate comes from cysteine. Our results highlight the ability of PDAC cells to adaptively rewire their metabolic pathways during knockdown of a key metabolic enzyme.

## Background

Pancreatic ductal adenocarcinoma (PDAC) is a devastating disease with < 10% of patients surviving beyond 5 years after diagnosis [1]. It is currently the fourth leading cause of cancer-related death in western countries and is expected to be the second leading cause by 2030 [2]. Currently, treatment options for pancreatic cancer patients include surgical resection, radiation therapy, and/or systemic chemotherapy [3, 4]. However, current therapies often fail because most patients are diagnosed at advanced stages. Given the poor outlook for PDAC patients, it is critical to improve our understanding of pancreatic cancer cells to design improved treatment strategies.

Reprogramming cancer metabolism is recognized as a hallmark of cancer [5]. Many tumor cells exhibit the “Warburg effect,” fermenting glucose to lactate even in the presence of abundant of oxygen [6, 7]. Indeed, pancreatic cancer cells rewire metabolism to utilize a wide range of nutrients including glucose, extracellular proteins, and various amino acids to support survival and proliferation [8–10]. Targeting metabolic pathways specifically upregulated in PDAC cells may be a promising direction for therapy [11].

Altered metabolism in cancer cells is supported in part by the expression of a specific isoform of pyruvate kinase (PK), a glycolytic enzyme which catalyzes the conversion of phosphoenolpyruvate (PEP) and ADP into pyruvate and ATP [12]. Pyruvate kinase has four isoforms (L, R, M1, and M2) encoded by two genes [13]. The *PKLR* gene encodes PKL and PKR, and the *PKM* gene encodes PKM1 and PKM2 through alternative splicing of exons 9 and 10, respectively [14]. PKL is mainly expressed in the liver, kidney, and small intestine and PKR in erythrocytes [15, 16]. PKM1 is mainly expressed in differentiated tissues such as muscle, heart, and brain, whereas PKM2 is expressed in various adult tissues and many proliferating cells, including embryonic and tumor cells [13, 15–17]. Generally, expression of PKM1 and PKM2 is mutually exclusive in a given cell type, and loss of PKM2 leads to compensatory expression of PKM1 [18]. PKM2 is highly expressed in a variety of human cancer cells, including pancreatic cancer cells [19]. PKM2 has been reported to promote proliferation, migration, invasion, and tumorigenesis in pancreatic cancer [20–23]. However, there are conflicting reports regarding the expression of PKM2 and overall patient survival: some studies show that PKM2 expression is associated with worse overall survival of pancreatic cancer patients [20, 24, 25], while others show improved overall survival [26], and still others show no effect on overall survival [27, 28]. A recent study demonstrated that expression of PKM1, but not PKM2, promotes small-cell lung cancer cell growth [29]. Other studies reported that PKM2 is dispensable for leukemia, liver cancer, colon cancer, lymphoma, lung cancer, and pancreatic cancer [30–34].

Given the controversial roles of pyruvate kinase in cancer, we investigated the function of PKM1/2 for pancreatic cancer in PDAC cells derived from a *Kras*^*G12D/-*^*; p53*^*-/-*^ pancreatic mouse model. Our results demonstrate that knockdown of PKM2 results in expression of PKM1 and does not affect pancreatic cancer cell proliferation. Additionally, pancreatic cancer cells are able to proliferate even with concurrent knockdown of both PKM1 and PKM2 isoforms. Further, they are able to produce pyruvate from glucose with knockdown of both PKM1 and PKM2 isoforms. We explored the contributions of alternative pathways to pyruvate production, such as the serine biosynthesis pathway and the sialic acid pathway during PKM1/2 knockdown. Knockout of phosphoglycerate dehydrogenase (PHGDH), the rate-limiting enzyme in the serine biosynthesis pathway, decreased pyruvate production from glucose in PKM1/2 knockdown cells. However, knockout of N-acetylneuraminate pyruvate lyase (NPL), which can convert phosphoenolpyruvate to pyruvate in the sialic acid pathway, did not decrease pyruvate production from glucose. Using multiple isotopically labeled precursors, we discovered that glucose contributes to only ~ 40% of intracellular pyruvate, and amino acid cysteine contributes ~ 20% of intracellular pyruvate in these cells. The relatively low contribution of glucose to pyruvate in combination with alternative sources for pyruvate generation may explain the minimal impact of PKM1/2 knockdown on pancreatic cancer cell proliferation.

## Methods

### Cell culture

A13M2-1 and A13M13 cell lines were derived from *Kras*^*G12D/-*^*; p53*^*-/-*^ pancreatic mouse tumor and contain doxycycline-inducible hairpins that target PKM2 or both PKM1 and PKM2, respectively [35, 36]. Cells were cultured in DMEM (Fisher Scientific, MT10017CV) without sodium pyruvate, supplemented with 10% fetal bovine serum (FBS), 1% penicillin and streptomycin (P/S), and 1% glutamine and cultured in a humidified incubator with 5% CO_2_ at 37 °C. For cells infected with inducible vectors, doxycycline aqueous solution (1 mg/ml as stock solution) was added at the time of plating at a final concentration of 1 μg/ml. Cells were passaged in doxycycline-containing media for 7 days to maximize knockdown of PKM1/2 before experiments were conducted.

### Cell proliferation assay

The cells were seeded at 25,000 per well in 6-well plates in normal cell growth medium. Cell counts were obtained using a Cellometer Auto T4 Cell Counter (Nexcelom).

### Gene knockout by CRISPR/Cas9 gene editing

We used CRISPR/Cas9-mediated genome editing to achieve gene knockout with lentivirus-mediated gene expression [37]. Dual-guide RNAs targeting PHGDH or NPL gene were designed by CRISPR DESIGN (http://crispr.mit.edu/) and set just before the protospacer adjacent motif (PAM), a DNA sequence immediately following the Cas9-targeted DNA sequence. All the specific target sequences were amplified and cloned into lentiviral vectors and verified by DNA sequencing. CRISPR gene editing plasmid vectors with gRNA and Cas9 co-expression were acquired from VectorBuilder. The VSVG plasmid was a gift from Bob Weinberg (Addgene plasmid # 8454; http://n2t.net/addgene:8454; RRID:Addgene 8454). The psPAX2 plasmid was a gift from Didier Trono (Addgene plasmid # 12260; http://n2t.net/addgene:12260; RRID:Addgene 12260). To produce lentivirus, HEK293T cells seeded in 10-cm plates were transfected with 10.0 μg lentivirus plasmids, 0.5 μg VSVG, and 5.0 μg psPAX2 plasmids. The following morning, fresh DMEM with 15% FBS and 1% P/S was added, and cells were grown for another 48 h to generate virus. For transduction with lentivirus, the A13M13 cells (1 × 10^5^ cells) were seeded in 10-cm plates and the supernatant of transfected HEK293T was collected and passed through 0.45 micron PVDF syringe filter. Five milliliters of the viral supernatant and 5 ml of fresh media were added to recipient A13M13 cell plates with polybrene (Fisher Scientific, TR1003G) at final concentration of 4 μg/ml. The cells were cultured for 24 h followed by adding fresh DMEM medium supplemented with 10% FBS and treated for 10 days with 10 μg/ml blasticidin (Fisher Scientific, A1113903) for selection. The blasticidin selected cells were then resuspended to a concentration of 5 cells/ml and seeded 1 cell/well on 96-well plates. Surviving clones were expanded and analyzed for successful gene knockout. Genomic DNA was extracted using DNeasy Blood and Tissue Kit (Qiagen) to check for successful gene editing. Dual gRNAs for PHGDH knockout plasmid vector are 5′-CGGGCTCAGCCCTCCGACCC-3′ and 5′-GGTGCTCCCTACCAAGCCGT-3′. Dual gRNAs for NPL knockout plasmid vector are 5′- GAGCGTCTCTGAACGTCGCC-3′ and 5′-CGTGGGAGCACTAAACGTGA-3′. To address PHGDH/NPL dual knockout, we designed a plasmid vector containing gRNAs for both PHGDH and NPL. The gRNAs are 5′-CGTGGGAGCACTAAACGTGA-3′ (targeting PHGDH) and 5′-CGGGCTCAGCCCTCCGACCC-3′ (targeting NPL). The sequence for scramble control plasmid with blasticidin selection maker is 5′-GCACTACCAGAGCTAACTCA-3′.

### Western blot analysis

Cell lysis and Western blot analysis were carried out according to standard protocols. The following dilutions of primary commercial antibodies were used as probes: 1:1000 dilution of anti-PKM1 (Cell Signaling Technology, 7067S), 1:1000 dilution of anti-PKM2 (Cell Signaling 4053S), 1:1000 dilution of β-actin (13E5) (Cell Signaling Technology, 4970S), 1:10000 dilution of anti-vinculin (E1E9V) (Cell Signaling Technology, 13901S), 1:1000 dilution of anti-GAPDH (Cell Signaling Technology, 5174S), and 1:1000 dilution of anti-PHGDH (Cell Signaling Technology, 13428S). Primary antibodies were diluted in 5% non-fat milk and incubated overnight at 4 °C. Secondary antibodies (Cell Signaling Technology, 7074S) were diluted in 5% non-fat milk at a dilution of 1:1000 and incubated at room temperature for 1 h.

### Metabolomic profiling and stable isotope labeling

For metabolite quantification, cells were seeded in triplicates (*n* = 3) in 6-well plates with DMEM supplemented with 10% FBS and 1% PS. For stable isotope labeling, media was switched to labeling media containing appropriate tracer, 25 mM [U-^13^C_6_]-glucose, 5 mM [U-^13^C_5_]-glutamine, 2 mM [U-^13^C_3_]-serine, 2 mM [U-^13^C_3_]-cysteine, 3.5 mM [U-^13^C_11_]-tryptophan, 7.2 mM [U-^13^C_4_]-threonine, 25 mM [1,2-^13^C_2_]-glucose, or 5 mM [5-^13^C_1_]-glutamine (all from Cambridge Isotopes Laboratories, Inc). Samples collected at *T* = 0 (unlabeled), 5 min, 30 min, 60 min, 120 min, and 24 h after starting the experiment. Metabolite extraction was performed as described previously [38]. Protein left from the extraction was dissolved in 0.2 M potassium hydroxide aqueous solution overnight, then quantified using Pierce BCA Protein Assay Kit (Fisher Scientific, PI23225). Dried metabolite extracts were resuspended in HPLC-grade water containing 1 μM 1,4-piperazinediethanesulfonic acid (PIPES; Sigma-Aldrich, P6757) as an internal standard. To normalize sample concentrations, samples were resuspended at volumes corresponding to their protein quantification values. For amino acid analysis, 20 μl of resuspended sample was added to 80 μl methanol and derivatized with 10 μl triethylamine and 2 μl benzylchloroformate. For pyruvate, lactate, and citrate analysis, 20 μl of resuspended sample was added to a mixture of 20 μl of 250 mM 3-nitrophenylhydrazine (3-NPH) in 50% methanol, 20 μl of 150 mM 1-ethyl-3-(3-dimethylaminopropyl) carbodiimide HCl (EDC) in methanol, and 20 μl of 7.5% pyridine in methanol and allowed to react at 30 °C for 30 min [39]. After this reaction, 16 μl of 2 mg/ml butylated hydroxytoluene (BHT) in methanol was quickly added to these solutions, which were then diluted with 104 μl of water. Samples with and without derivatization were transferred to HPLC vials for analysis.

LC-MS/MS analysis was performed with ion-pairing reverse phase chromatography using an Ascentis Express column (C18, 5 cm × 2.1 mm, 2.7 μm, Sigma-Aldrich) for separation and a Waters Xevo TQ-S triple quadrupole mass spectrometer operated in negative mode as mass analyzer. LC parameters were described previously [38]. Peak processing was performed in MAVEN [40]. Isotope labeling data was corrected for the natural abundance of different isotopes using IsoCor [41]. Heat maps were generated using Cluster [42].

### Statistical analysis

Statistical analysis was performed using a two-tailed Student’s *t* test.

## Results

### PKM2 and PKM1/2 knockdown do not decrease pancreatic cancer cell proliferation

**Fig. 1:**
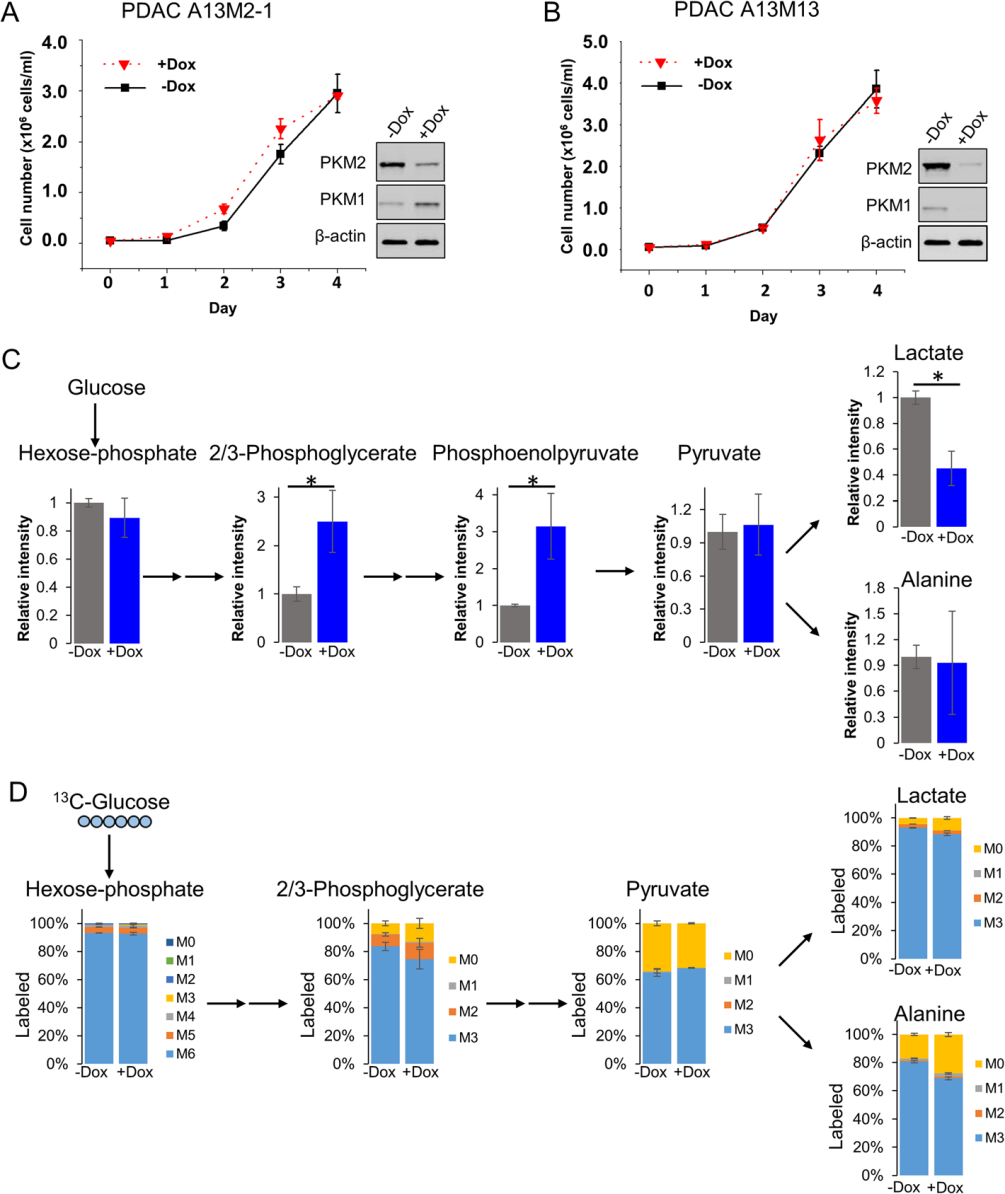
PKM2 and PKM1/2 knockdown do not decrease proliferation of pancreatic cancer cells. **a**, **b** PDACs proliferate at the same rate with (+Dox) or without (−Dox) PKM1/2 knockdown. Proliferation was assessed by counting cell numbers in triplicates for 4 days following 7 days of vehicle (−Dox) or 1 μg/ml doxycycline (+Dox) treatment to maximize PKM2 or PKM1/2 knockdown. Western blot confirms PKM1/2 knockdown in PDACs after 7 days of vehicle (−Dox) or doxycycline (+Dox) treatment. **c** Relative intracellular metabolites levels are represented by peak intensities and are displayed relative to −Dox averages. Values are the average of three biological replicates. Error bars represent standard deviation. Statistically significant differences (*p* value < 0.05) are marked with asterisks (*). **d** ^13^C-glucose labeling of intracellular metabolites for 24 h in A13M13 PDAC cells with vehicle (−Dox) or PKM1/2 knockdown (+Dox). The *y*-axis for all graphs is the percent labeling of indicated ^13^C-isotopologue

To study the role of pyruvate kinase in pancreatic cancer proliferation, we characterized PDAC cell lines (A13M2-1 and A13M13) derived from a *Kras*^*G12D/-*^*; p53*^*-/-*^ mouse pancreatic tumor [35]. A13M2-1 contains a doxycycline-inducible hairpin that knocks down ~ 85% of PKM2 expression after 7 days of doxycycline treatment. Knockdown of PKM2 does not arrest proliferation of PDAC cells (Fig. 1a). Consistent with previous reports [34], knockdown of PKM2 in PDACs induced PKM1 expression (Fig. 1a). Since PKM1 expression may compensate for PKM2 knockdown, we further knocked down both PKM1 and PKM2 expression in the A13M13 cell line, which contains a doxycycline-inducible short hairpin RNA (shRNA) that knocks down ~ 85% of both PKM1 and PKM2 after 7-day doxycycline treatment (Fig. 1b and Additional file 1: Figure S1A). Unexpectedly, knockdown of both M1 and M2 isoforms still does not affect PDAC cell proliferation (Fig. 1b). To ensure that PKL or PKR expression was not induced after PKM1/2 knockdown, we probed the expression of PKL/R in PDAC cells. Western blot results indicated that there is no PKL/R expressed in A13M13 cells (Additional file 1: Figure S1B). We also measured intracellular metabolites levels following knockdown of both M1 and M2 isoforms in A13M13 PDAC cells (Fig. 1c). Knockdown of PKM1/2 greatly elevated upstream intermediates of glycolysis, such as 2/3-phosphoglycerate (2/3-PG) and phosphoenolpyruvate (Fig. 1c). This is consistent with decreased glycolytic flux through PK, indicating that PK is the rate-limiting step in glycolysis during PKM knockdown. Pyruvate levels were unchanged, but downstream intermediate lactate was significantly decreased with PKM1/2 knockdown. We further performed ^13^C-glucose labeling experiments to determine how glucose is processed through different metabolic pathways. Surprisingly, PDACs with PKM1/2 knockdown are able to generate proportionally similar amounts of ^13^C-labeled pyruvate (Fig. 1d). Approximately 60% of pyruvate and 90% of lactate and alanine were labeled in PDAC cells with PKM1/2 knockdown, indicating that pyruvate can still be made from glucose despite targeting a key glycolytic enzyme.

### Serine biosynthesis pathway is upregulated in PDACs following PKM1/2 knockdown

**Fig. 2:**
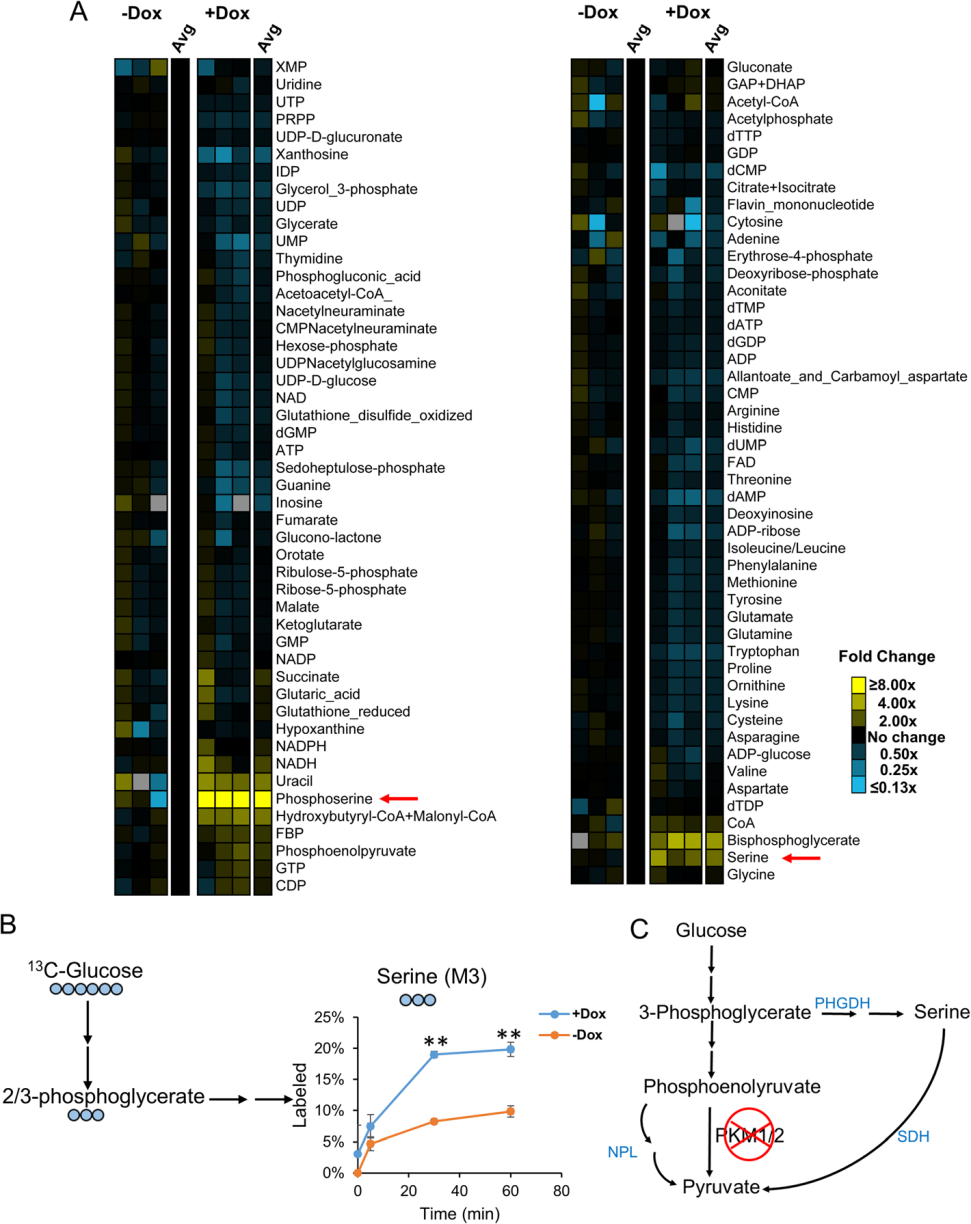
Pancreatic cancer cells upregulate the serine biosynthesis pathway following PKM1/2 knockdown. **a** Pool sizes of intracellular metabolites in PDAC cells treated with (+Dox) or without (−Dox) PKM1/2 knockdown were detected using ultrahigh performance liquid chromatography tandem mass spectrometry (UPLC-MS/MS). **b** ^13^C-Glucose flux to serine is upregulated in A13M13 PDAC cells with PKM1/2 knockdown (+Dox). Statistically significant differences (*p* value < 0.01) are marked with asterisks (**). **c** Metabolic pathways that may generate pyruvate from upstream glycolytic intermediates during PKM knockdown. NPL, N-acetylneuraminate pyruvate lyase; SDH, serine dehydratase

While PKM1/2 knockdown does not decrease PDAC cell proliferation (Fig. 1b), it does impact cellular metabolism. PDACs displayed different intracellular metabolic profiles upon PKM1/2 knockdown (Fig. 2a). As expected, PKM1/2 knockdown causes an accumulation of glycolytic intermediates upstream of pyruvate, including fructose 1,6-bisphosphate (FBP), 2/3-PG, and PEP. In addition, serine biosynthesis pathway intermediates phosphoserine and serine were vastly elevated. In contrast, many other amino acids were decreased in PKM1/2 knockdown cells (Fig. 2a). Further investigation with ^13^C-glucose labeling studies showed that glucose flux to serine is upregulated in PDACs with PKM1/2 knockdown (Fig. 2b). Since serine can be converted to pyruvate via the action of serine dehydratase [43], we postulated that the serine biosynthesis pathway may be one way to circumvent PKM1/2 knockdown in pancreatic cancer cells (Fig. 2c).

### PHGDH knockout depletes intracellular serine and decreases pyruvate production

**Fig. 3:**
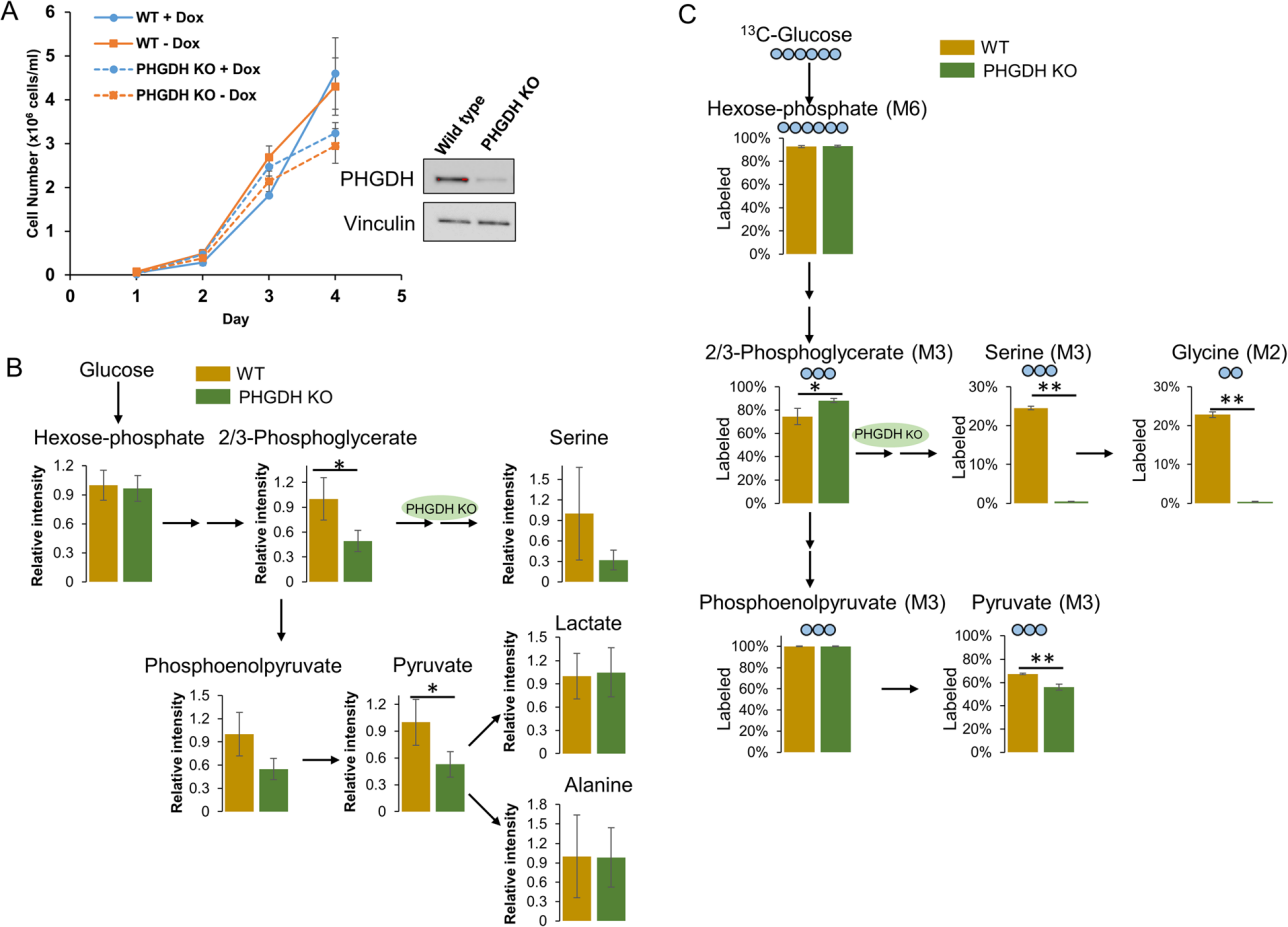
PHGDH knockout depletes intracellular serine, slightly decreases pyruvate generation, but does not affect cell proliferation. **a** Proliferation rates of wild-type A13M13 (PHGDH WT) or PHGDH knockout (PHGDH KO) populations of PDACs with vehicle (−Dox) or PKM1/2 knockdown (+Dox). Cell counts were measured daily (*n* = 3) after vehicle or doxycycline treatment. Western blot confirms PHGDH knockout in PDACs. **b** Relative intracellular metabolites levels are represented by peak intensities and are displayed relative to WT metabolite intensity averages. Cells are collected after 7 days of doxycycline treatment. Values are the average of three biological replicates. Error bars represent standard deviation. **c** ^13^C-Glucose labeling of intracellular metabolites for 24 h in WT and PHGDH KO PDAC clone P24 cells with PKM1/2 knockdown. The *y*-axis for all graphs is the percent labeling of indicated full labeled ^13^C-isotopologue. Experiments were performed in triplicates and all data are displayed as the mean values ± standard error. Statistically significant differences (*p* value) are marked with asterisks (*p < 0.05, **p < 0.01)

It is possible that cells bypass PKM1/2 knockdown and convert glucose to pyruvate through the serine biosynthesis pathway (Fig. 2c). To explore this possibility, we targeted the serine biosynthesis pathway by deleting phosphoglycerate dehydrogenase (PHGDH), the rate-limiting enzyme in this pathway. PHGDH converts NAD^+^ and 3-phosphoglycerate to NADH and 3-phosphohydroxypyruvate [44–46]. We deleted PHGDH using CRISPR/Cas9 gene editing technology. We successfully generated PHGDH knockout (KO) PDAC populations and single cell clones, as confirmed by Western blotting (Additional file 1: Figure S2F) and DNA sequencing (Additional file 1: Figure S3). We find that PHGDH KO does not affect PDAC population cell proliferation following PKM1/2 knockdown (Fig. 3a and Additional file 1: Figure S2A–E). We also measured the levels of serine in PHGDH KO clone cells. As expected, knocking out PHGDH significantly decreased serine levels (Fig. 3b). We also find that PHGDH KO indeed led to reduced pyruvate levels (Fig. 3b). Interestingly, PHGDH KO resulted in decreased levels of upstream glycolytic intermediates 2/3-PG and PEP (Fig. 3b), suggesting that targeting the serine biosynthesis pathway may downregulate glycolysis. We further performed ^13^C-glucose labeling and confirmed that knockout of PHGDH abolished serine and glycine production from ^13^C-glucose as indicated by loss of M3 and M2 labeling in serine and glycine, respectively (Fig. 3c). Additionally, the fraction of M3-labeled pyruvate from ^13^C-glucose was slightly decreased, indicating that the serine biosynthesis pathway indeed contributes to pyruvate production during PKM1/2 knockdown.

### PHGDH/NPL dual knockout does not impact cell growth rate and pyruvate generation

**Fig. 4:**
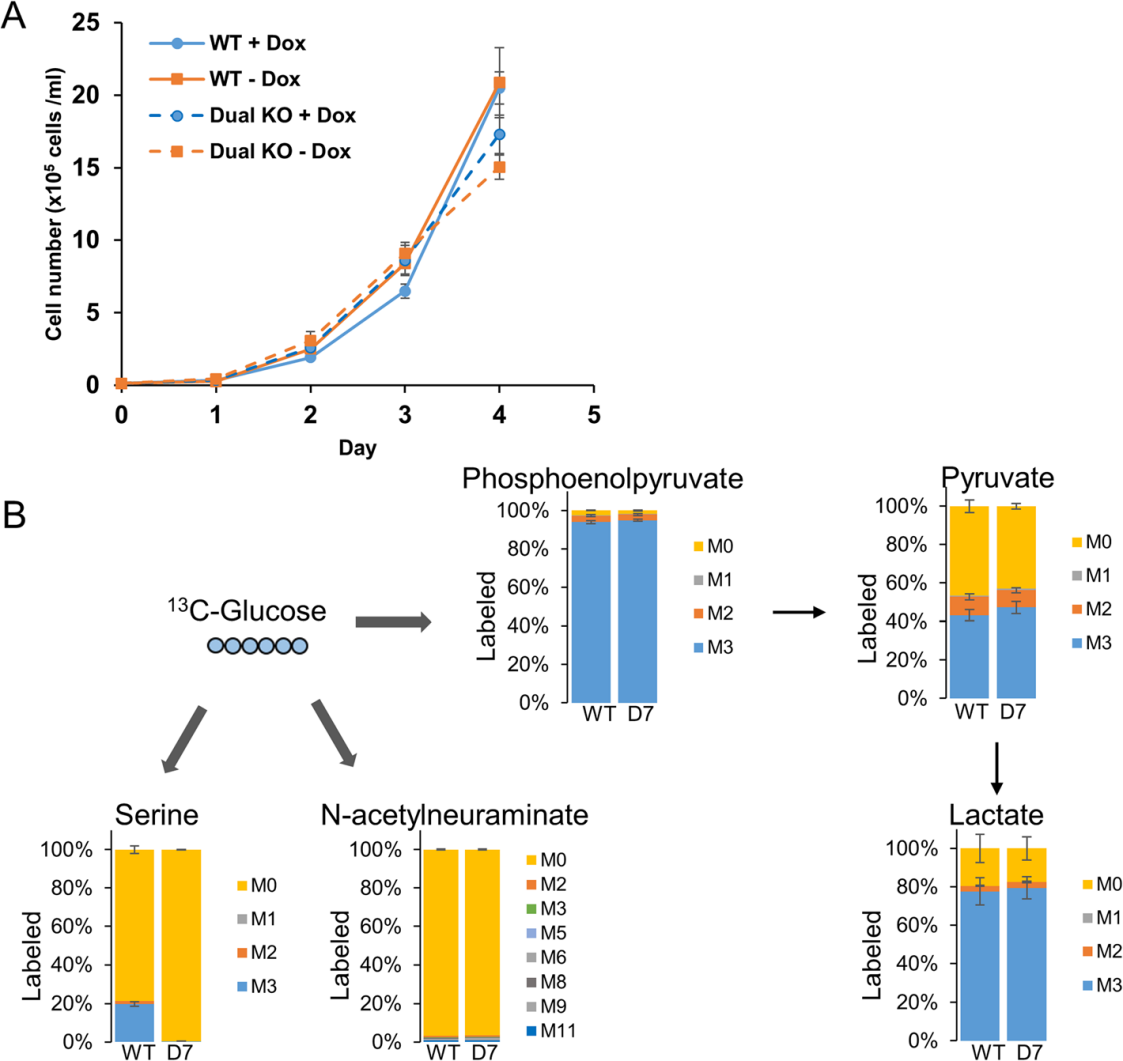
PHGDH and NPL dual KO does not impact growth rate and pyruvate generation from glucose. **a** Average proliferation rates of 3 PHGDH/NPL dual knockouts (dual KO) clones or wild-type PDACs with vehicle (−Dox) or PKM1/2 knockdown (+Dox). Cell counts were measured daily (*n* = 3). **b** ^13^C-Glucose labeling of intracellular metabolites for 60 min in wild-type (WT) or PHGDH/NPL dual knockout clone D7 cells with PKM1/2 knockdown for 60 min. The *y*-axis for all graphs is the percent labeling of indicated ^13^C-isotopologue. Experiments were performed in triplicates, and all data are displayed as the mean values ± standard error

Since ^13^C-glucose still contributes to a significant fraction of labeled pyruvate in PHGDH KO cells with PKM1/2 knockdown (Fig. 3c), we investigated whether the sialic acid pathway contributes to producing pyruvate from upstream glycolytic intermediates (Fig. 2c). N-acetylneuraminate synthase (NANS) produces N-acetylneuraminic acid (sialic acid) from N-acetylmannosamine and PEP, while N-acetylneuraminate pyruvate lyase (NPL) catalyzes the degradation of sialic acid into N-acetylmannosamine and pyruvate [47, 48]; hence, this pathway could potentially bypass pyruvate kinase to convert glucose-derived phosphoenolpyruvate into pyruvate. To test this possibility, we generated NPL knockout PDAC cells by using CRISPR/Cas9. NPL knockout PDAC clones can proliferate as well as wild-type PDAC cells following PKM1/2 knockdown (Additional file 1: Figure S4A). Since it is possible that cells use the serine biosynthesis pathway for pyruvate generation during NPL knockout, we further generated PHGDH/NPL dual knockout (dual KO) PDAC cells by using CRISPR/Cas9 (Additional file 1: Figure S5), as confirmed by sequencing (Additional file 1: Figure S6). We find that dual KO cells do not suffer loss of proliferative ability with PKM1/2 knockdown (Fig. 4a). We further investigated the metabolic impacts in PHGDH/NPL dual KO cells using ^13^C-glucose labeling. Glucose can still be converted into pyruvate in wild-type and dual KO cells following PKM1/2 knockdown (Fig. 4b). Although N-acetylneuraminate is labeled by ^13^C-glucose after 24 h (Additional file 1: Figure S1C), it was not labeled in either wild-type or PHGDH/NPL dual KO cells after 60 min, indicating that ^13^C-glucose did not go through the sialic acid pathway to generate labeled pyruvate in this experiment.

### Other sources of pyruvate

**Fig. 5:**
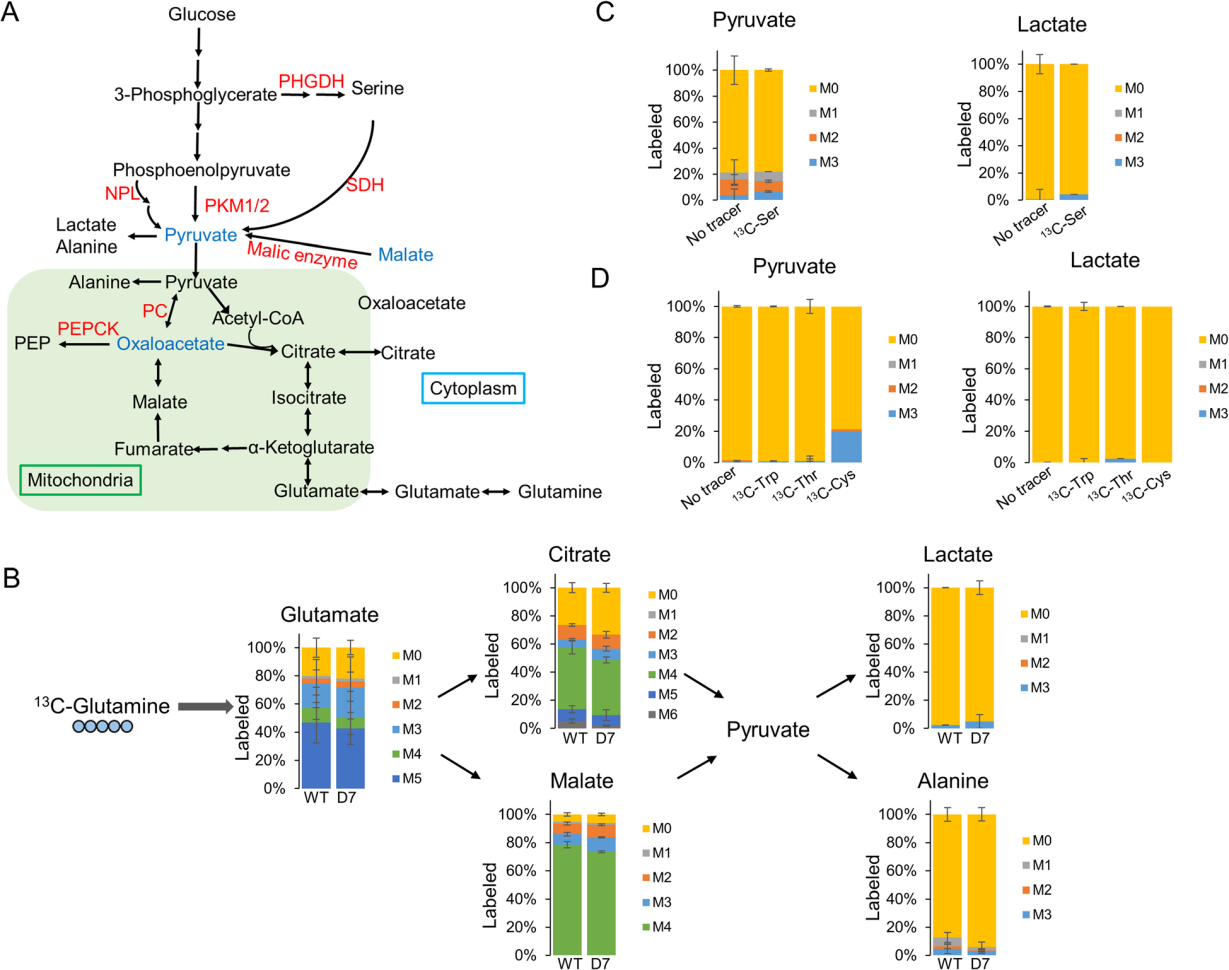
Investigation of additional pathways for pyruvate generation. **a** Metabolic pathways that may generate pyruvate from other intermediates during PKM1/2 knockdown. PEPCK, phosphoenolpyruvate carboxykinase; PC, pyruvate carboxylase. **b** ^13^C-Glutamine labeling of intracellular metabolites for 60 min in wild-type (WT) and PHGDH/NPL dual KO D7 PDAC cells with PKM1/2 knockdown. **c** ^13^C-Serine labeling of intracellular metabolites for 60 min in PHGDH/NPL dual KO D7 PDAC cells with PKM1/2 knockdown. **d** ^13^C-Tryptophan, ^13^C-threonine, and ^13^C-cysteine labeling of intracellular metabolites for 60 min in PHGDH/NPL dual KO D7 PDAC cells with PKM1/2 knockdown. The *y*-axis for all graphs is the percent labeling of indicated ^13^C-isotopologue. Experiments were performed in triplicates and all data are displayed as the mean values ± standard error. Ser, serine; Trp, tryptophan; Thr, threonine; Cys, cysteine

A significant fraction of pyruvate remains unlabeled even after incubation with ^13^C-glucose for 24 h in both wild-type and PHGDH KO cell lines with or without PKM1/2 knockdown (Fig. 3c). This suggests that pyruvate can be generated from sources other than glucose in these cell lines. Potential pathways that can generate pyruvate during PKM1/2 knockdown are shown in Fig. 5a. Studies of pyruvate metabolism demonstrate that anaplerotic pyruvate entry by pyruvate carboxylase (PC) in the mitochondria will generate oxaloacetate, which can produce phosphoenolpyruvate through mitochondrial phosphoenolpyruvate carboxykinase (PEPCK-M). PEP is then transported out of the mitochondrial matrix by an anion transporter into the cytosol. Cytosolic malate also can be converted to pyruvate through malic enzyme. To determine whether PC, PEPCK-M, or malic enzyme actively contribute to pyruvate production in PDAC cells, we characterized ^13^C-glutamine labeling in dual KO cells. While a large proportion of citrate (~ 60%) and malate (~ 80%) were labeled from ^13^C-glutamine, the downstream metabolites of pyruvate—lactate and alanine—remained virtually unlabeled (Fig. 5b). These results suggest that glutamine-derived TCA cycle intermediates are not converted into pyruvate in these cells. We further extended the labeling time and showed that pyruvate, alanine, and lactate are not labeled by ^13^C-glutamine even after 24 h (Additional file 1: Figure S7A). Pyruvate can also be produced from several other amino acids. Serine can be converted to pyruvate by serine dehydratase. Threonine can be cleaved to yield glycine [49], which is converted to serine by serine hydroxymethyltransferase (SHMT) and then converted to pyruvate by serine dehydratase. Tryptophan catabolism via the kynurenine pathway yields alanine [50], which can be converted to pyruvate via transamination. Cysteine may also be catabolized to yield pyruvate and inorganic bisulfite [51]. We further investigated pyruvate generation from amino acids by using ^13^C-serine, ^13^C-tryptophan, ^13^C-threonine, and ^13^C-cysteine tracers. Surprisingly, we find that ^13^C-serine has negligible contribution to pyruvate and lactate labeling (Fig. 5c and Additional file 1: Figure S7D). On the other hand, cysteine contributes to an unexpectedly large proportion (~ 20%) of pyruvate in dual KO cells (Fig. 5d). Cysteine has not been previously reported as a major source of pyruvate production in PDAC cells. WT PDAC cells also produce labeled pyruvate (~ 10%) from ^13^C-cysteine (Additional file 1: Figure S8), indicating that these cells generally produce pyruvate from cysteine. The lower production of pyruvate from cysteine in WT cells (~ 10%) compared to PHGDH/NPL dual KO cells (~ 20%) is likely due to the dual KO cells having impaired pyruvate production through other pathways (e.g., serine biosynthesis pathway) and requiring a larger proportion of pyruvate production from cysteine. Other amino acids did not significantly contribute to the production of pyruvate (Fig. 5d).

**Fig. 6:**
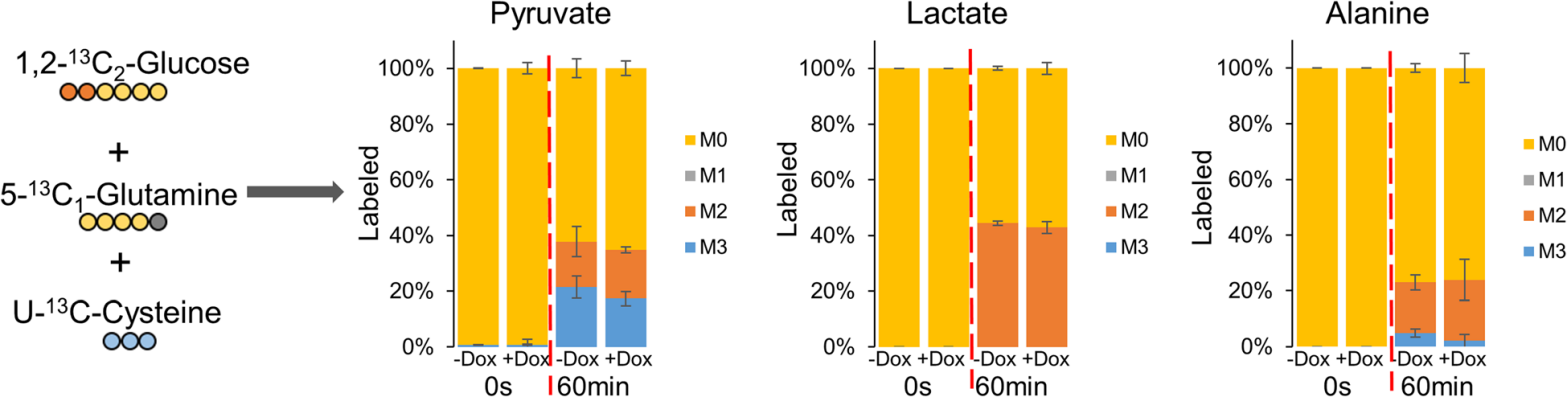
Labeling with multiple tracers shows that ~ 20% of pyruvate is generated from cysteine. Multi-tracer of 1,2-^13^C_2_-glucose, 5-^13^C_1_-glutamine, and ^13^C-cysteine labeling of intracellular metabolites in PHGDH/NPL dual knockout D7 PDAC cells following with vehicle (−Dox) or PKM1/2 knockdown (+Dox). The *y*-axis for all graphs is the percent labeling of indicated ^13^C-isotopologue. Experiments were performed in triplicates and all data are displayed as the mean values ± standard error

To better understand the sources of pyruvate, we performed a multi-tracer experiment in PHGDH/NPL dual KO cells. Cells were cultured with 1,2-^13^C-glucose, 5-^13^C-glutamine, and U-^13^C-cysteine for 60 min (Fig. 6). Approximately 40% of pyruvate is generated from glucose, as 20% of labeled pyruvate (M2) and 20% of unlabeled pyruvate (M0) comes from 1,2-^13^C-glucose. Consistent with the lack of alanine and lactate labeling from ^13^C-glutamine (Fig. 5b), pyruvate, alanine, and lactate were not labeled by ^13^C-glutamine in this multi-tracer experiment. Interestingly, 20% of labeled pyruvate (M3) comes from ^13^C-cysteine.

## Discussion

In this study, we demonstrate that pancreatic cancer cells can rewire their metabolism and continue to proliferate during PKM1/2 knockdown. PKM2 in cancer has been studied since the early twenty-first century, but its roles remain controversial. High expression of PKM2 is correlated with poor prognosis in pancreatic cancer patients [20, 24, 25], and suppression of PKM2 expression resulted in decreased cell survival [20, 52]. On the contrary, our results, like other published studies investigating PKM2 function in pancreatic cancer [53, 54], showed that knockdown of PKM2 expression has no effect on PDAC cell proliferation. Similar results were observed in other cancer models, including breast cancer [55], hepatocellular carcinoma [32], colon cancer [33], and leukemia [31]. Differences between our results and published reports may be due to incomplete knockout of PKM1/2 in our cells; despite ~ 85% depletion in PKM1/2 expression, a small fraction of PKM1/2 may be enough for maintaining pyruvate production. Complete knockout of PKM1/2 did not yield any surviving clones in multiple experiments, indicating a small amount of PKM1/2 is likely essential for in vitro survival of these cells. The impact of pyruvate kinase on cancer cell proliferation is likely dependent on the context of each cancer cell, including genetic makeup, mutations, and the microenvironment.

We find that downregulation of PKM1/2 expression does not impact pyruvate production in PDAC cells. However, we do observe rewiring of metabolism following PKM1/2 knockdown: there is accumulation of upstream glycolytic intermediates, consistent with previous reports [30, 52, 56]. We further find that PKM1/2 knockdown causes an increase in serine biosynthesis intermediates. This may be due to an accumulation of upstream glycolytic intermediates causing increased flux through the serine biosynthesis pathway. Knockout of PHGDH, the rate-limiting enzyme in the serine biosynthesis pathway, decreases pyruvate production as indicated by both pyruvate levels (Fig. 3b) and pyruvate labeling from ^13^C-glucose (Fig. 3c). Thus, our data suggests that the serine biosynthesis pathway contributes to the production of pyruvate from glucose during PKM1/2 knockdown. However, since addition of ^13^C-serine to the media does not result in pyruvate labeling (Fig. 5c), serine imported from the media does not appear to be directly converted into pyruvate. It is possible that flux through the serine biosynthesis pathway may have a regulatory effect on pyruvate production, since serine is an allosteric activator of PKM2 [57]. Despite reports that suppression of PHGDH can impair cancer cell proliferation [45, 46, 58], we find that PHGDH deletion does not attenuate cell proliferation in PDAC cells. This may be related to the fact that ^13^C-glucose-derived serine comprises only a small fraction of overall serine; most serine remained unlabeled after 24 h (Fig. 3c) and presumably originated from the culture media. This data agrees with another study that shows the majority of intracellular serine is obtained extracellularly in T cells [59]. Finally, we note that PHGDH KO results in accumulation of nucleotide intermediates (PRPP, UMP, UTP, UDP, dUMP, dTMP, dTTP, and ATP), TCA cycle intermediates (α-ketoglutarate, fumarate, and citrate/isocitrate), and many amino acids (Additional file 1: Figure S9), indicating that PHGDH deletion causes glucose flux to be shunted to nucleotides, the TCA cycle, and amino acid biosynthesis.

During PKM1/2 knockdown, cell growth is supported in part by alternative pathways that generate pyruvate, including breakdown of exogenous amino acids. We explored the breakdown of cysteine, serine, glutamine, tryptophan, and threonine into pyruvate. Cysteine is a key sulfur-containing semi-essential amino acid which plays important functions in redox homeostasis, protein function, and metabolism [60]. Interestingly, our results show that about 20% of pyruvate came from ^13^C-cysteine (Fig. 6). This is an unexpectedly high fraction, since cysteine has not been previously reported as a major source of intracellular pyruvate in PDAC cells. WT PDAC cells also generate pyruvate from ^13^C-cysteine (Additional file 1: Figure S8), indicating that this is a general pathway of cysteine production in these cells. The higher fraction of cysteine-derived pyruvate in PHGDH/NPL dual KO cells (~20%; Fig. 6) compared to WT cells (~ 10%; Additional file 1: Figure S8) may be due to the dual KO cells having impaired pyruvate production through other pathways (e.g., serine biosynthesis pathway) and hence requiring a larger proportion of pyruvate generation from cysteine. We also observed a trend toward decreased cysteine pools in the WT cells upon PKM knockdown (Fig. 2a) which may be indicative of cysteine utilization to produce pyruvate, supporting the notion that cysteine is a major source of pyruvate in PDAC cells. The labeled pyruvate from ^13^C-cysteine is not further converted to lactate or alanine, as indicated by the absence of M3 lactate labeling in either metabolite (Fig. 6). Since lactate is produced in the cytosol, and alanine is predominantly produced in the mitochondria, this data may suggest that cysteine is converted to pyruvate within another cellular compartment that is distinct from either the cytosol or mitochondria. The dependence on cysteine is a potential metabolic adaption for pyruvate generation in PDAC cells. Glutamine is another potential source of pyruvate that we investigated. As the most abundant amino acid in plasma, upregulated glutamine metabolism has been found in several cancer types [11]. Once transported into mitochondria, glutamine serves as a carbon source for the production of fatty acids, nucleotides, and TCA cycle intermediates, and up to 60% of glutamine has been reported to be used for generating lactate and alanine. In our study, glutamine does not contribute to pyruvate generation in PDACs (Fig. 5b and Additional file 1: Figure S7A), despite their dependence on glutamine for proliferation (Additional file 1: Figure S10). Tryptophan and threonine can also be converted to pyruvate, but this was not observed in our cells (Fig. 5d, Additional file 1: Figure S7B and S7C). Thus, our results show that ~ 40% of pyruvate is generated from glucose and ~ 20% of pyruvate is generated from cysteine. For our labeling experiments, we used DMEM formulated without pyruvate and dialyzed FBS that does not contain low molecular weight compounds like amino acids. We further checked the media by LCMS and confirmed that both dialyzed FBS and DMEM contain negligible amounts of pyruvate (Additional file 1: Figure S11). It is possible that the remaining unlabeled portion of pyruvate is produced from amino acids released by degradation of serum proteins in the media, as macropinocytosis and extracellular protein scavenging has been observed in pancreatic cancer cells [61–63]. Since the cells produce pyruvate from cysteine, cystine in the media—which can be imported via xCT and subsequently converted to cysteine intracellularly—may also contribute to the unlabeled portion of pyruvate.

## Conclusions

Pancreatic cancer cells rewire their metabolism during pyruvate kinase knockdown. The serine biosynthesis pathway enables some conversion of glucose to pyruvate during pyruvate kinase knockdown; however, since direct conversion of serine to pyruvate was not observed, it appears that the serine pathway contributes to pyruvate generation through a potentially regulatory mechanism. We also find that a surprisingly large percentage of intracellular pyruvate comes from cysteine. Our study reveals the resilience of PDAC cells to pyruvate kinase knockdown, underscoring the metabolic flexibility of these cells to overcome environmental perturbation and maintain proliferation.

## Supplementary information

**Additional file 1: Supplementary Figures.**

## Data Availability

All data generated or analyzed during this study are included in this article.

## Abbreviations

2/3-PG: 2/3-Phosphoglycerate
3-NPH: 3-Nitrophenylhydrazines
BHT: Butylated hydroxytoluene
Cys: Cysteine
Dox: Doxycycline
EDC: 1-Ethyl-3-(3-dimethylaminopropyl) carbodiimide HCl
FBP: Fructose 1,6-bisphosphate
Gly: Glycine
LC-MS/MS: Liquid chromatography tandem mass spectrometry
NANS: N-Acetylneuraminate synthase
NPL: N-Acetylneuraminate pyruvate lyase
PC: Pyruvate carboxylase
PDAC: Pancreatic ductal adenocarcinoma
PEP: Phosphoenolpyruvate
PEPCK-M: Mitochondrial phosphoenolpyruvate carboxykinase
PHGDH: Phosphoglycerate dehydrogenase
PIPES: Piperazine-*N*,*N*-bis (2-ethanesulfonic acid)
PKM2: Pyruvate kinase M2 isoform
P/S: Penicillin and streptomycin
SDH: Serine dehydratase
Ser: Serine
SHMT: Serine hydroxymethyltransferase
Thr: Threonine
Trp: Tryptophan
