# Supplementary material for: Cysteine catabolism and the serine biosynthesis pathway support pyruvate production during pyruvate kinase knockdown in pancreatic cancer cells
Source: Cancer Metab. 2019 Dec 30;7:13. doi: 10.1186/s40170-019-0205-z (PMC6937848; doi:10.1186/s40170-019-0205-z)
Supplement: Supplementary file 1 — Additional file 1: Supplementary Figures. [file 40170_2019_205_MOESM1_ESM.docx]

**Additional File**

**Cysteine catabolism and the serine biosynthesis pathway support pyruvate production during pyruvate kinase knockdown**

**in pancreatic cancer cells**

Lei Yu^1^, Shao Thing Teoh^1^, Elliot Ensink^1^, Martin P. Ogrodzinski^1,2^, Che Yang^1^, Ana I. Vazquez^3,4^, Sophia Y. Lunt^1,5*^

^1^Department of Biochemistry and Molecular Biology, Michigan State University, East Lansing, MI, USA

^2^Department of Physiology, Michigan State University, East Lansing, MI, USA

^3^Department of Epidemiology and Biostatistics, Michigan State University, East Lansing, MI, USA

^4^The Institute for Quantitative Health Science and Engineering, Michigan State University, East Lansing, MI, USA

^5^Department of Chemical Engineering and Materials Science, Michigan State University, East Lansing, MI, USA

*Correspondence:

Sophia Y. Lunt, Ph.D.

Biochemistry Building

603 Wilson Rd Room 522A

Michigan State University

East Lansing, MI 48824, USA

Phone: 517-432-4886

Email: sophia@msu.edu

**
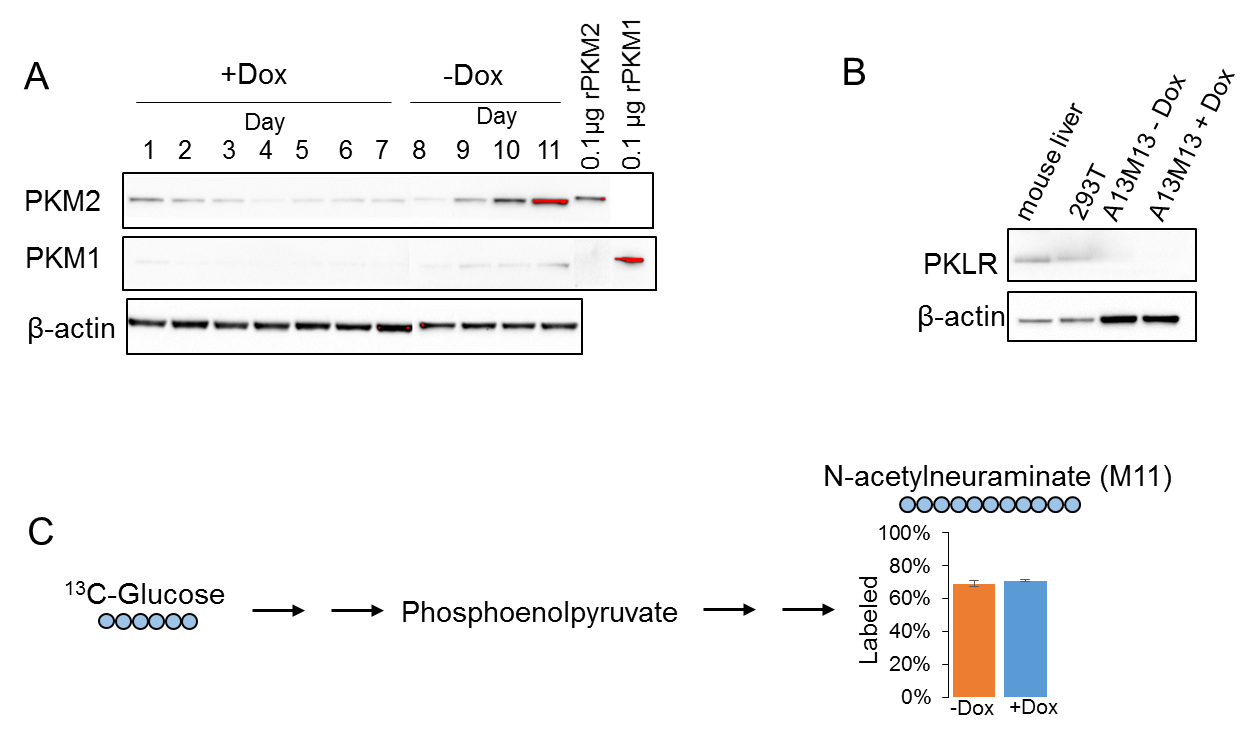
**

**Figure S1**. (A) The effect of doxycycline (Dox) treatment on pyruvate kinase isoform expression in pancreatic cancer cells (PDACs) was assessed by western blot analysis. Cells were treated with 1 μg/ml doxycycline (+Dox) or vehicle (-Dox) for the indicated number of days. Recombinant PKM1 or PKM2 lysate were included as control samples that express only PKM1 or PKM2. (B) No PKL/R is expressed in A13M13 cells. (C) ^13^C- glucose labeling of intracellular N-acetylneuraminate in PDAC cells with vehicle (−Dox) or PKM1/2 knockdown (+Dox). The y-axis for all graphs is the percent labeling of indicated ^13^C-isotopologue.

**
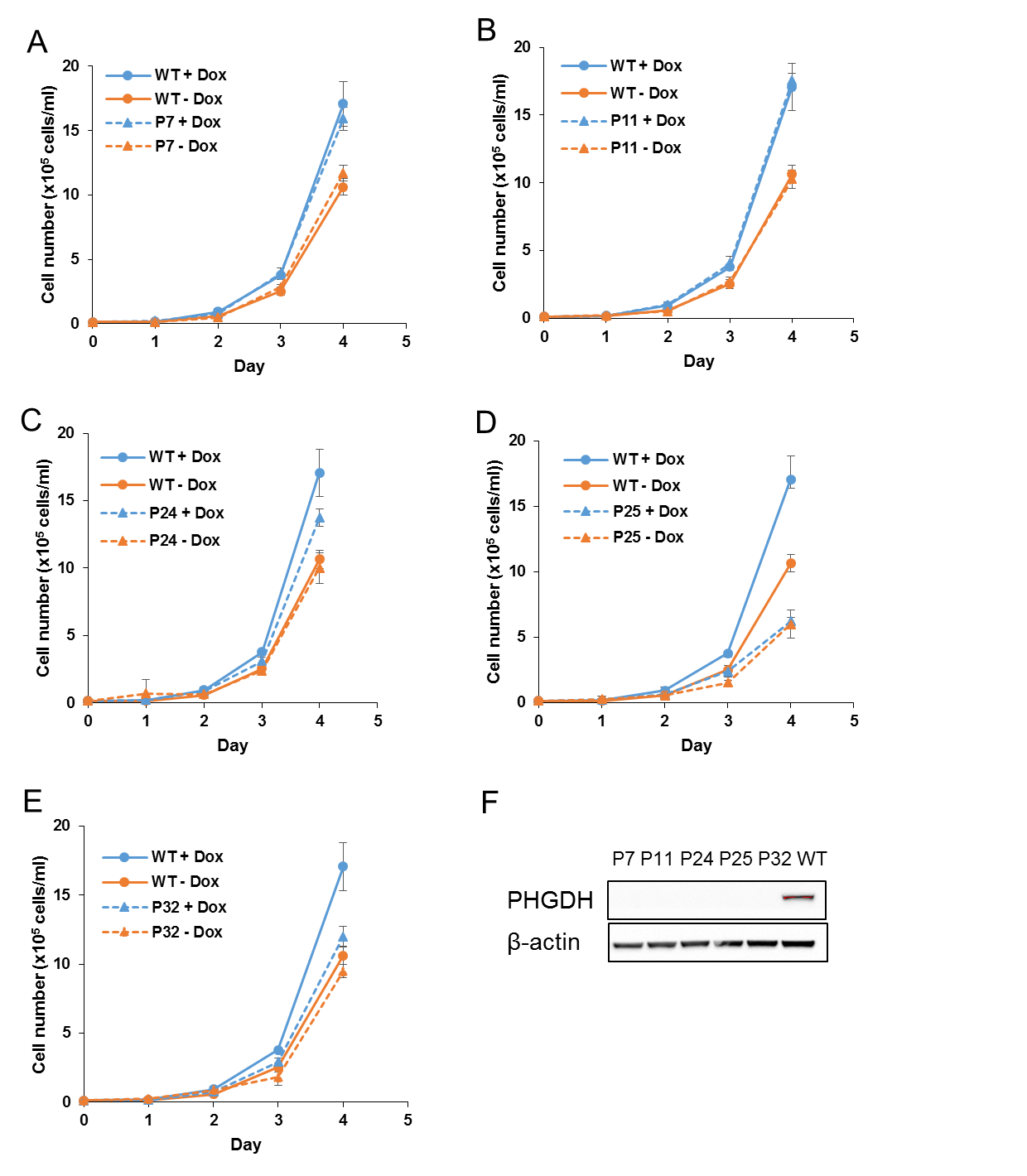
**

**Figure S2**. (A-E) Proliferation rates of PHGDH CRISPR knockouts of PDAC cell lines with vehicle (−Dox) or PKM1/2 knockdown (+Dox). P7, P11, P24, P25 and P32 are PHGDH knockout clones. WT is wild type PDAC cells. Cell counts were measured daily (n = 3). (F) Western blot results for wild type and PHGDH knockout PDACs. Protein extraction is performed after cells were treated with doxycycline or vehicle for 7 days.


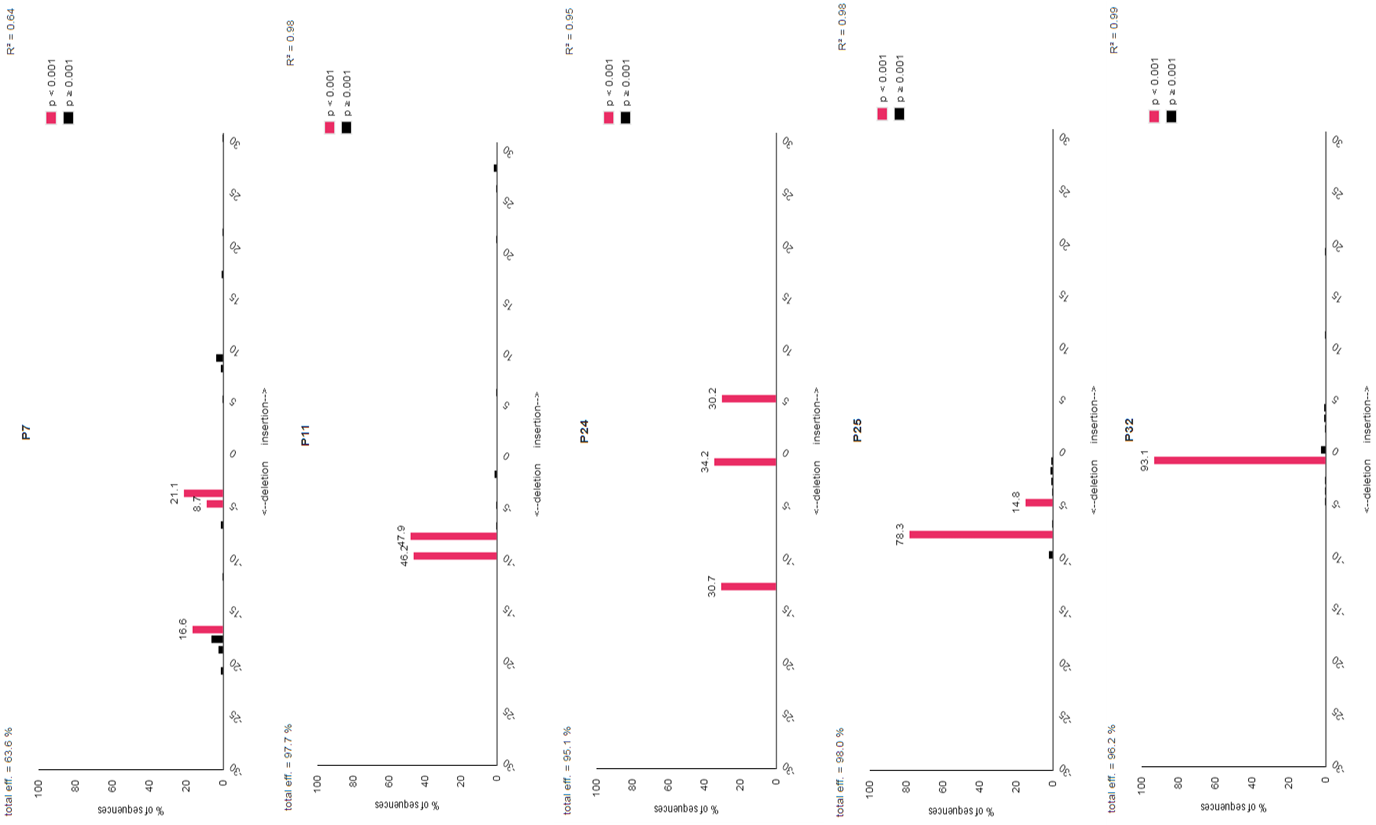


**Figure S3**. Sequencing confirmation of PHGDH knockout clones. About 800bp region enclosing the CRISPR target site was sequenced and analyzed with TIDE (Tracking of Indels by Decomposition) (http://tide.nki.nl/). TIDE generates indel spectra showing the predicted indels in a mixed population of sequences, as well as the proportion of each sequence. The R^2^ value indicates the total proportion of sequences that were successfully analyzed. p-values are calculated for each indel to indicate confidence in the existence of that indel, and indels with high confidence (p-value < 0.001) are highlighted in red.


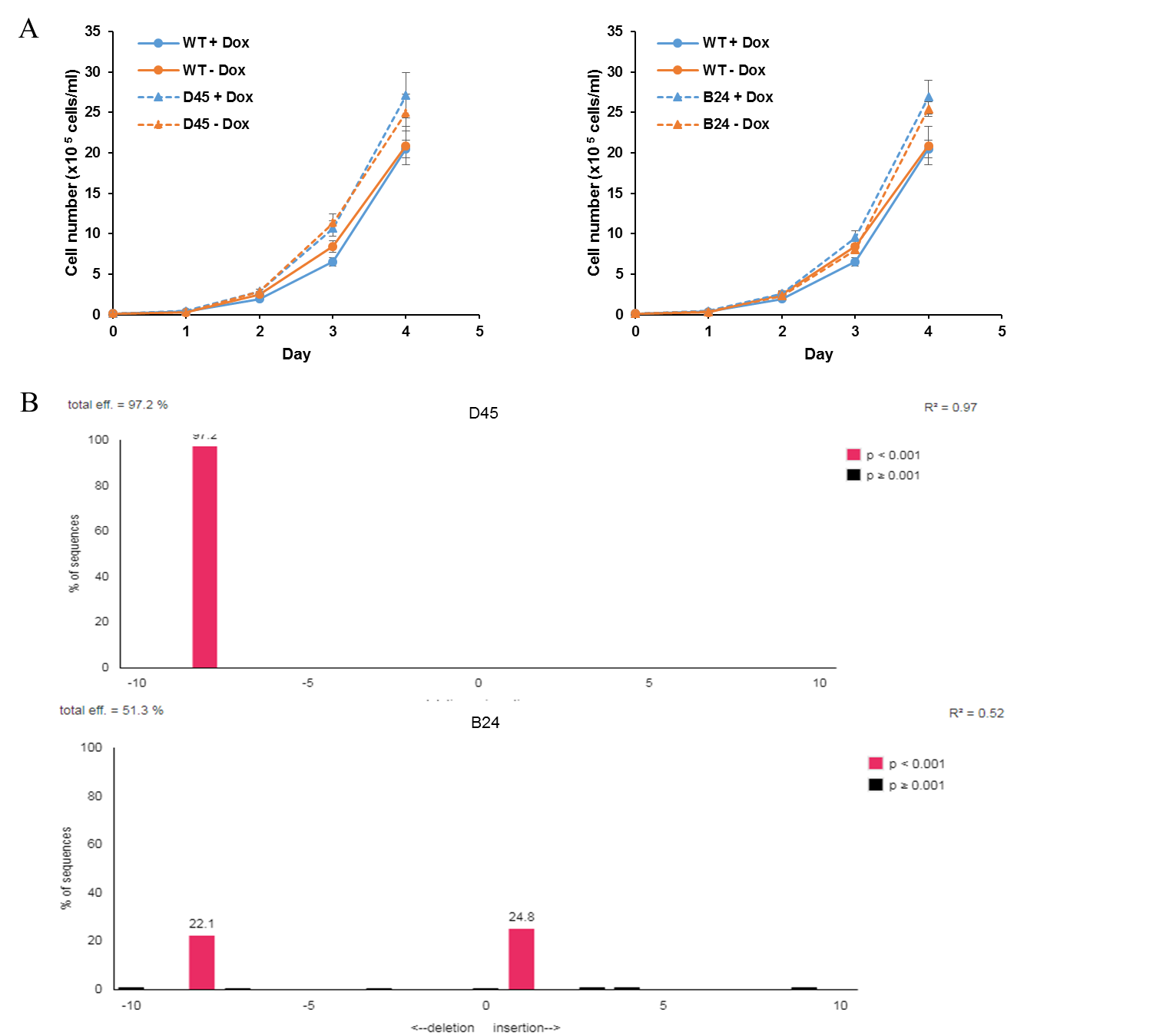


**Figure S4**. Proliferation rates (A) and sequence data (B) of NPL knockout clones D45 and B24 were performed as described above.


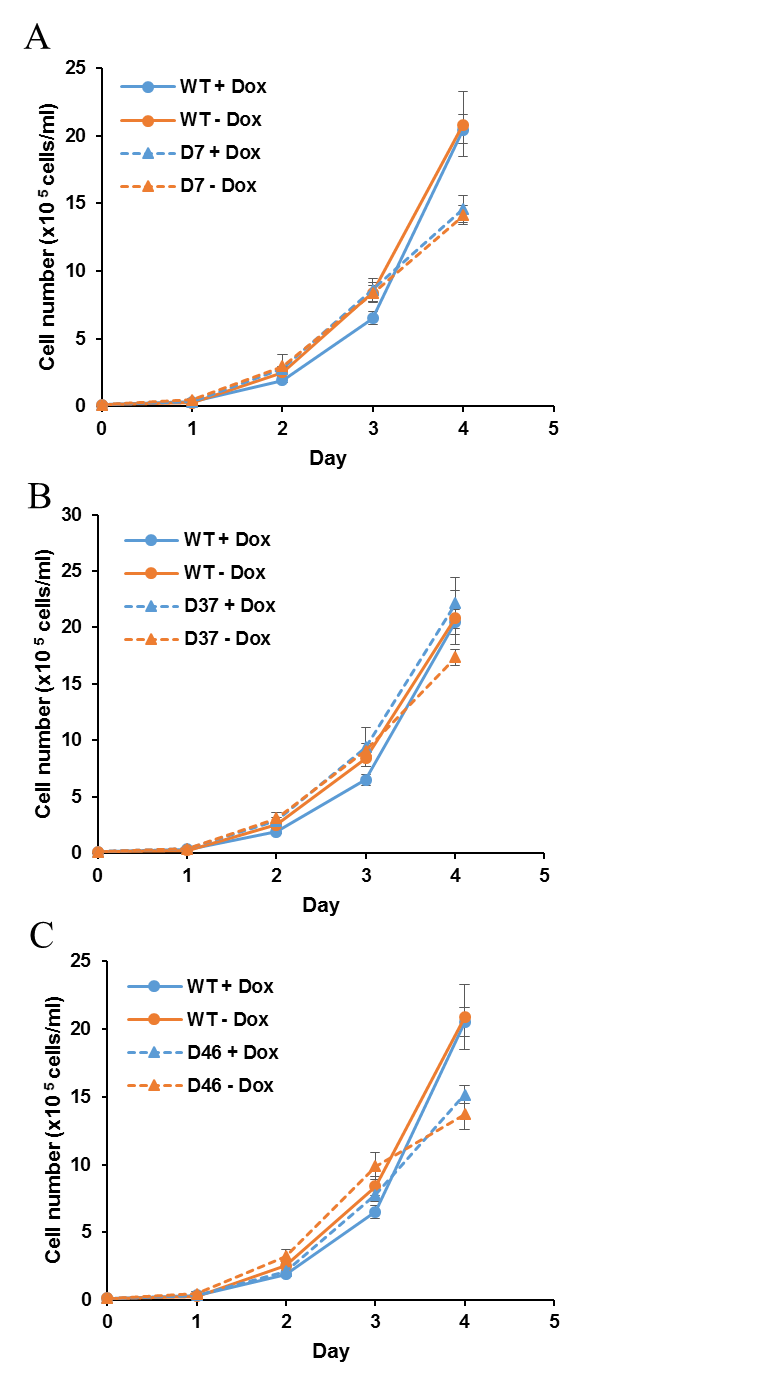


**Figure S5**. (A-C) Proliferation rates of PHGDH/NPL dual knockouts of PDAC cell lines with vehicle (−Dox) or PKM1/2 knockdown (+Dox). Cell counts were measured daily (n = 3).


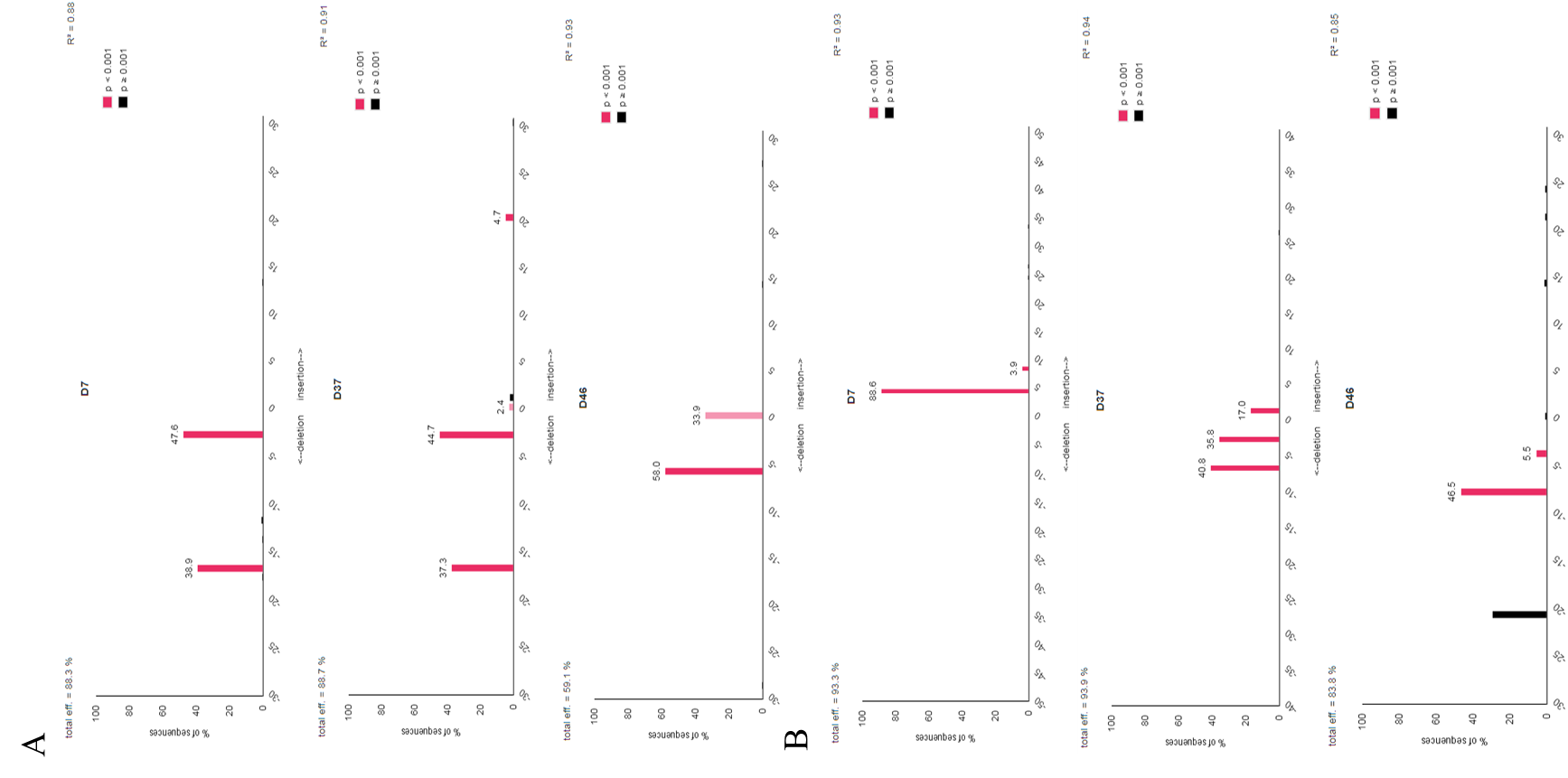


**Figure S6**. Sequencing confirmation of PHGDH/NPL dual knockout clones. (A) NPL knockout confirmation, (B) PHGDH knockout confirmation.


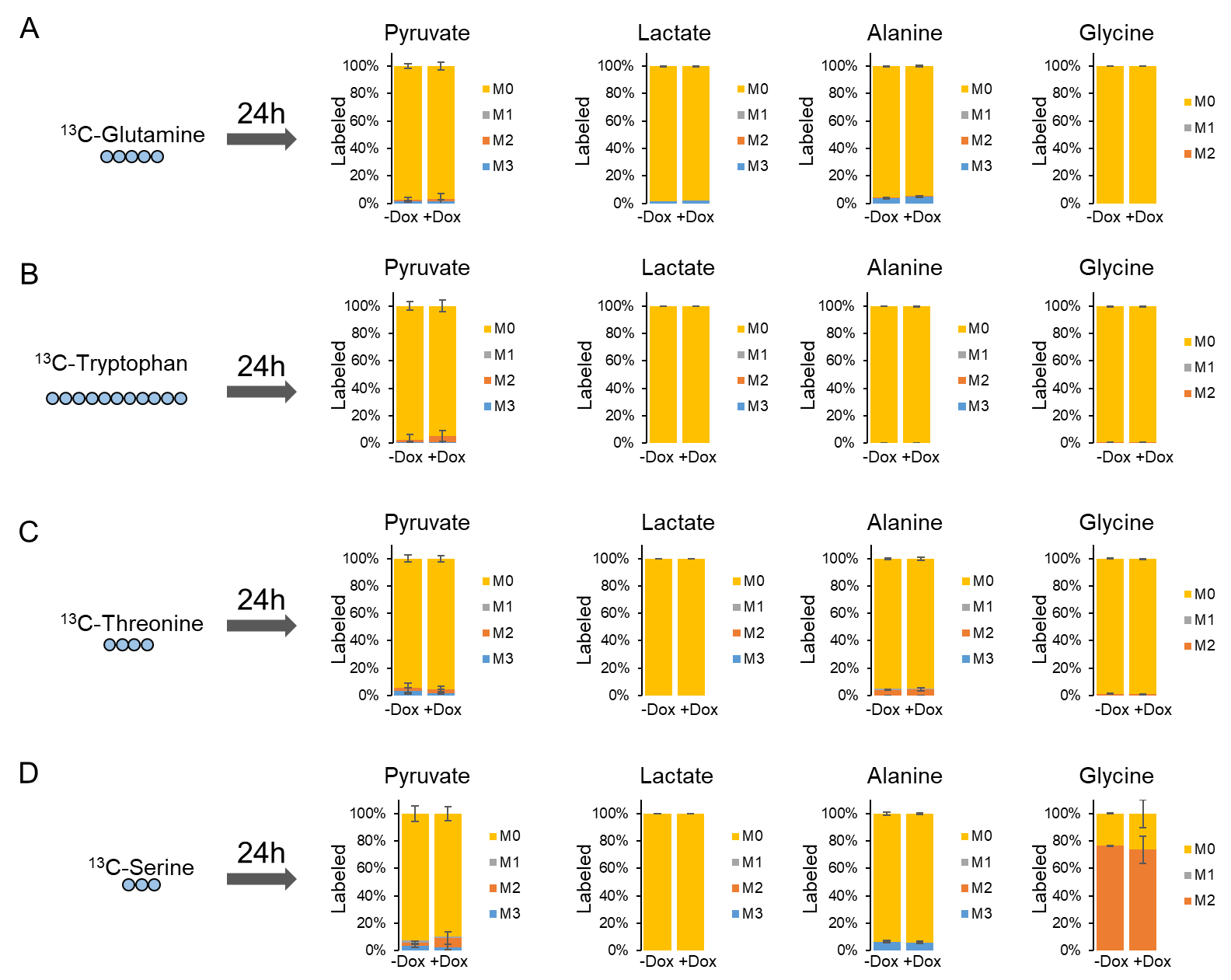


**Figure S7**. (A) U-^13^C-glutamine, (B) U-^13^C-tryptophan, (C) U-^13^C-threonine, and (D) U-^13^C-serine labeling of intracellular metabolites in PHGDH/NPL dual KO D7 PDAC cells with vehicle (−Dox) or PKM1/2 knockdown (+Dox). The y-axis for all graphs is the percent labeling of indicated ^13^C-isotopologue. All data are displayed as means of triplicates.


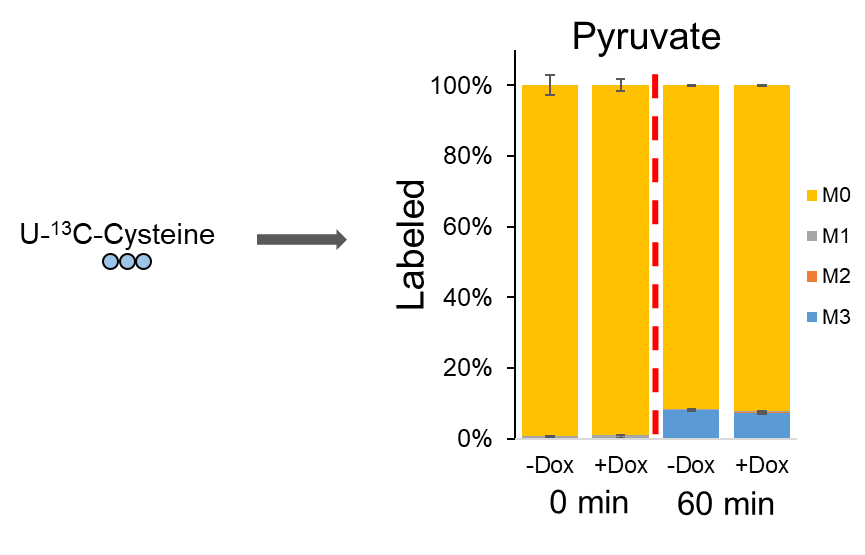


**Figure S8**. Labeling with ^13^C-Cysteine generates ~10% labeling of pyruvate in PDAC cells following vehicle (-Dox) or PKM1/2 knockdown (+Dox). The y-axis is the percent labeling of indicated ^13^C-isotopologue. Experiments were performed in triplicates, and all data are displayed as the mean values ± standard error.


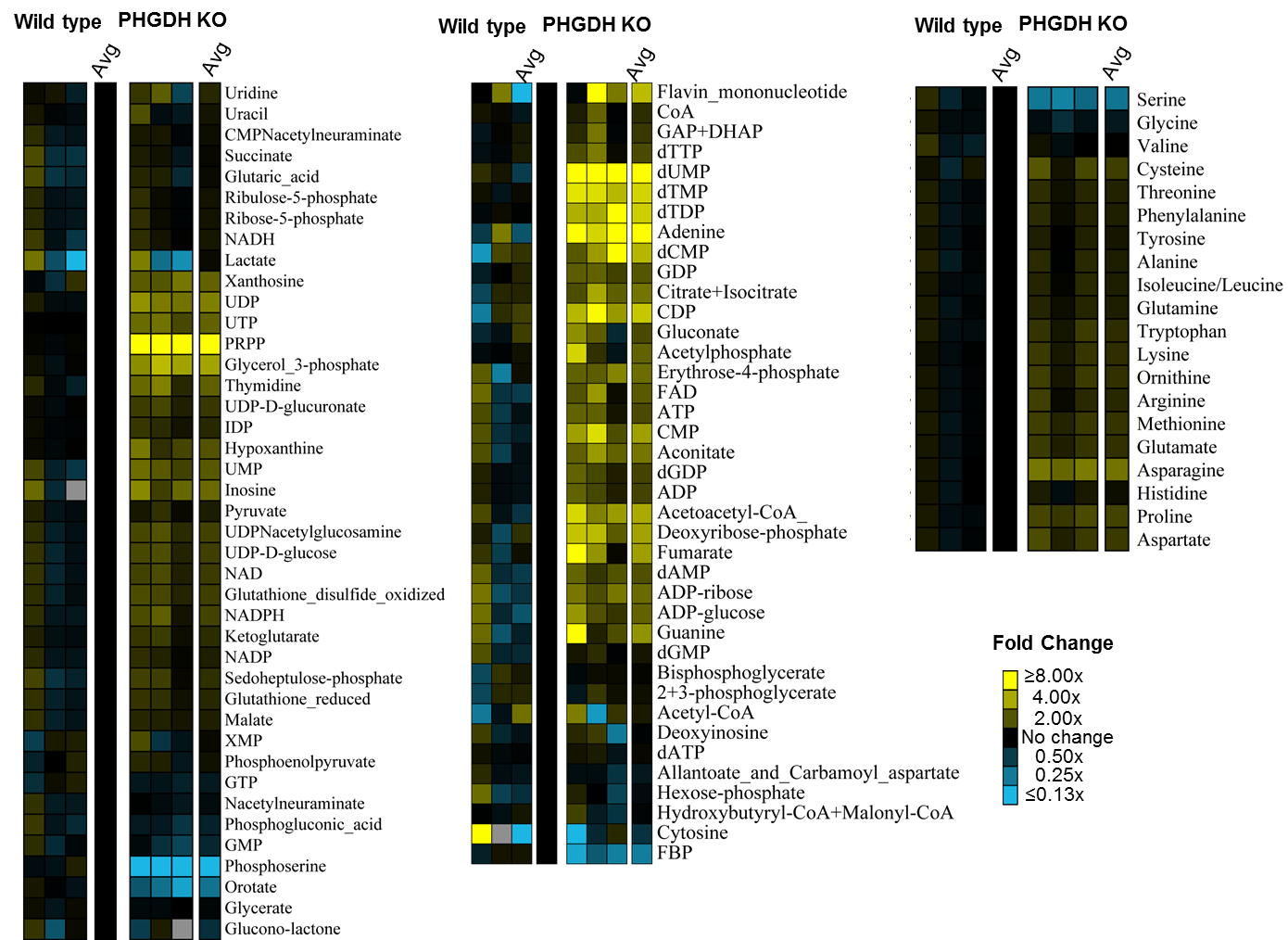


**Figure S9**. Intracellular metabolites in wild type and PHGDH knockout PDAC populations with PKM1/2 knockdown were measured using UPLC-MS/MS.


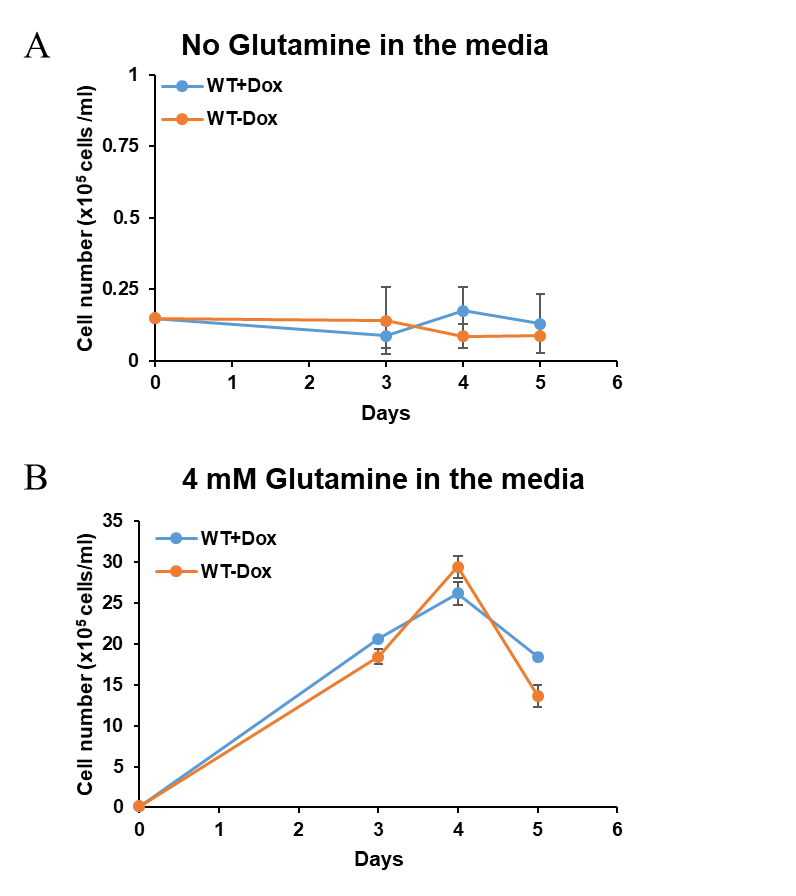


**Figure S10**. PDACs require glutamine for cell proliferation. Proliferation rates of A13M13 PDAC cells without (A) or with 4 mM glutamine (B) with vehicle (−Dox) or PKM1/2 knockdown (+Dox). All data are displayed as means of triplicates.


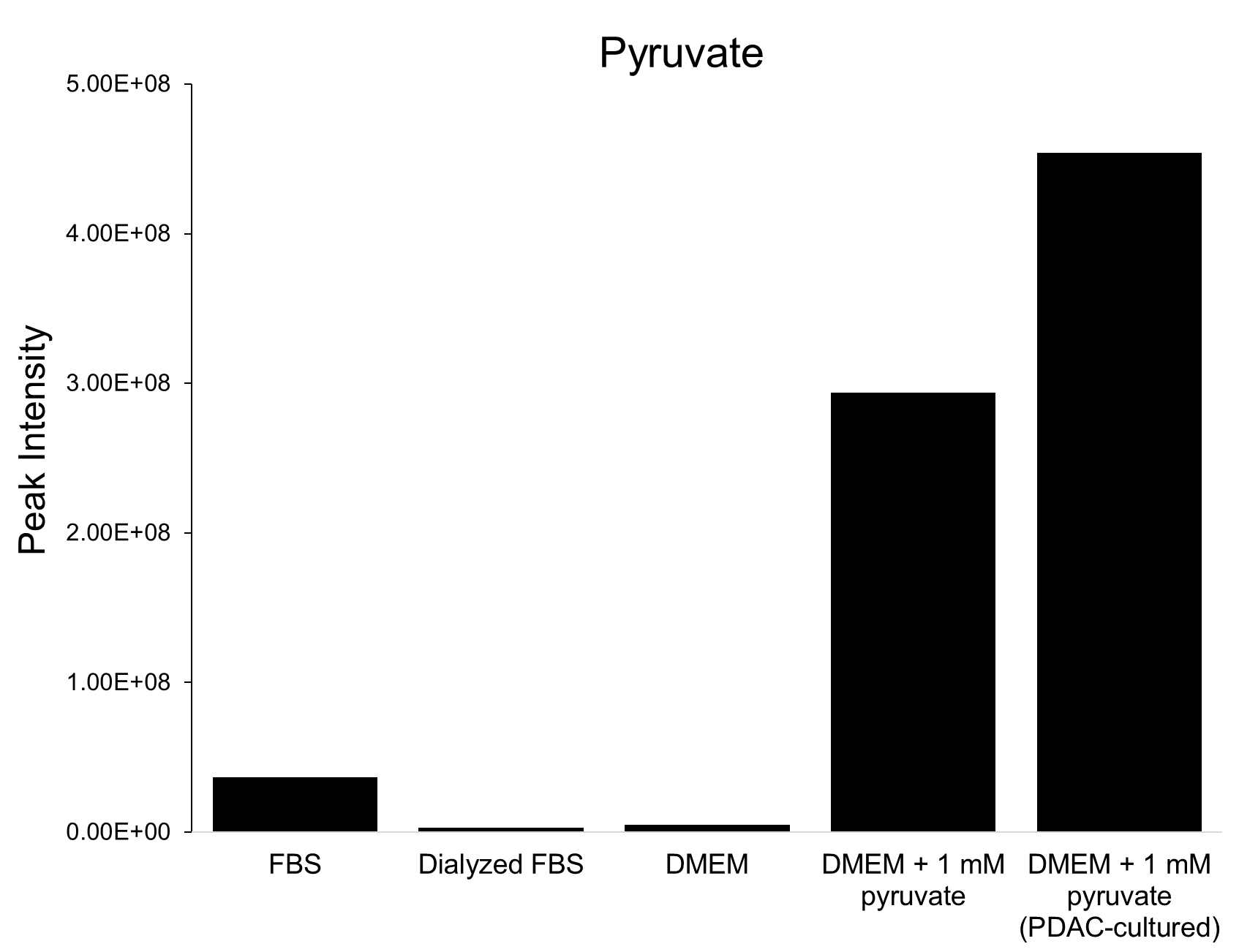


**Figure S11**. **Abundance of pyruvate in serum and media.** FBS (fetal bovine serum), dialyzed FBS (Sigma, F0392), DMEM (Fisher Scientific, MT10017CV), DMEM + 1 mM pyruvate (Fisher Scientific, MT10013CV). WT A13M13 PDAC cells were incubated in DMEM + 1 mM pyruvate on 3 separate plates. Media was collected from each plate and pooled together for analysis.
